# Effectiveness of the Fungal Metabolite 3-*O*-Methylfunicone towards Canine Coronavirus in a Canine Fibrosarcoma Cell Line (A72)

**DOI:** 10.3390/antibiotics11111594

**Published:** 2022-11-11

**Authors:** Claudia Cerracchio, Valentina Iovane, Maria Michela Salvatore, Maria Grazia Amoroso, Hiba Dakroub, Marina DellaGreca, Rosario Nicoletti, Anna Andolfi, Filomena Fiorito

**Affiliations:** 1 Department of Veterinary Medicine and Animal Production, University of Naples Federico II, 80137 Naples, Italy; 2 Department of Agricultural Sciences, University of Naples Federico II, 80055 Portici, Italy; 3 Department of Chemical Sciences, University of Naples Federico II, 80126 Naples, Italy; 4 Institute for Sustainable Plant Protection, National Research Council, 80055 Portici, Italy; 5 Istituto Zooprofilattico Sperimentale del Mezzogiorno, Unit of Virology, Department of Animal Health, 80055 Portici, Italy; 6 Council for Agricultural Research and Economics, Research Centre for Olive, Fruit and Citrus Crops, 81100 Caserta, Italy; 7 BAT Center-Interuniversity Center for Studies on Bioinspired Agro-Environmental Technology, University of Naples Federico II, 80055 Portici, Italy

**Keywords:** secondary metabolites, *Talaromyces pinophilus*, canine coronavirus, A72 cells, virus yield, viral nucleocapsid protein, aryl hydrocarbon receptor

## Abstract

Canine coronavirus (CCoV), an alphacoronavirus, may cause self-limiting enteric disease in dogs, especially in puppies. The noteworthy plasticity of coronaviruses (CoVs) occurs through mutation and recombination processes, which sometimes generate new dangerous variants. The ongoing SARS-CoV-2 pandemic and the isolation of a novel canine–feline recombinant alphacoronavirus from humans emphasizes the cross-species transmission ability of CoVs. In this context, exploring antiviral compounds is essential to find new tools for fighting against CoVs infections. Fungi produce secondary metabolites, which are often developed as antibiotics, fungicides, hormones, and plant growth regulators. Previous examinations of benzo-γ-pyrone 3-*O*-methylfunicone (OMF), obtained from *Talaromyces pinophilus*, showed that it reduces the infectivity of hepatitis C virus and bovine herpesvirus 1. Based on this evidence, this study evaluated the antiviral ability of OMF against CCoV infection in a canine fibrosarcoma (A72) cell line. During CCoV infection, a non-toxic dose of OMF markedly increased features of cell viability. Moreover, OMF induced a significant reduction in virus yield in the presence of an intense downregulation of the viral nucleocapsid protein (NP). These findings occurred in the presence of a marked reduction in the aryl hydrocarbon receptor (AhR) expression. Taken together, preliminary findings suggest that OMF inhibiting AhR shows promising activity against CCoV infection.

## 1. Introduction

Canine coronavirus (CCoV) belongs to the genus of alphacoronaviruses and is the etiological agent of enteric infections in dogs. Genotype II of canine coronavirus (CCoV-II) is characterized by high morbidity and low mortality [1,2,3], but an extremely virulent CCoV-IIa pantropic strain has been detected in Italian outbreaks of fatal disease in puppies, which spreads in extraintestinal tissues causing multi-systemic infections [2,4,5,6,7]. Recent studies also reported CCoV spillover from wild animals into human patients. In fact, a new CCoV has been isolated in Malaysia from a child patient hospitalized for pneumonia between 2017 and 2018, which has been identified via genome sequencing and called CCoV-HuPn-2018 [8]. Moreover, the HuCCoV_Z19Haiti, a novel CCoV, has been isolated from a medical team member presenting fever and malaise after travel to Haiti [9]. These cases are evidence of the direct consequence of the recombination and mutation tendency of coronavirus (CoV) genomes, which allows them to overcome natural barriers that usually prevent cross-species transmission, and to adapt to new species [1,2,10]. In fact, molecular mechanisms influence the adaptation, transmissibility, host/tissue tropism, and pathogenicity of viruses causing the production of novel strains.

The recent pandemic of COVID-19 due to severe acute respiratory syndrome-coronavirus-2 (SARS-CoV-2) has turned the spotlight on CoVs that are able to mutate into new and more dangerous strains. Hence, in the current scenario of emerging pathogenic coronaviruses with pandemic potential similar to SARS-CoV2, there is intense investigational activity focused on finding novel antivirals. Natural products offer a rich source of potential materials for the identification and development of novel drugs. In particular, fungal metabolites are regarded for their valued properties, such as structural variability, original mechanism of action, and bioactivities [11,12]. Funicones, as well as related compounds, constitute a homogeneous group of fungal polyketides possessing remarkable biological activities which have promoted their consideration as drug possibilities [13,14]. Among them, 3-*O*-methylfunicone (OMF) has shown significant antifungal, antiproliferative, and pro-apoptotic activities [15], as well as potential antiviral properties, towards hepatitis C virus (HCV) [16] and bovine herpesvirus type-1 (BoHV-1) [17].

Based on these observations, the potential antiviral effect of OMF against CCoV infection in canine fibrosarcoma cells (A72) was investigated after assessing the non-toxic in vitro dose of this compound.

## 2. Results

### 2.1. 3-O-Methylfunicone (OMF)

**Figure 1:**
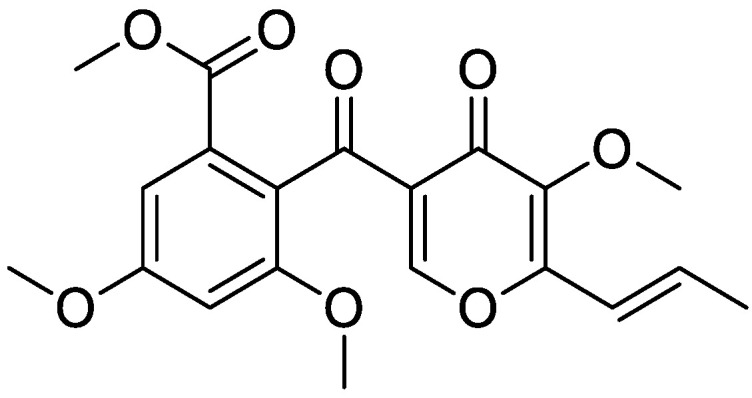
Chemical structure of 3-*O*-methylfunicone (OMF).

OMF (Figure 1) was purified from a chloroform extract of a liquid culture of isolate LT6 of *Talaromyces pinophilus*, using a chromatographic process (see Section 4.1). NMR spectroscopic characteristics were the same as the authentic standard compound [18].

### 2.2. OMF Increased Cell Viability during CCoV Infection

**Figure 2:**
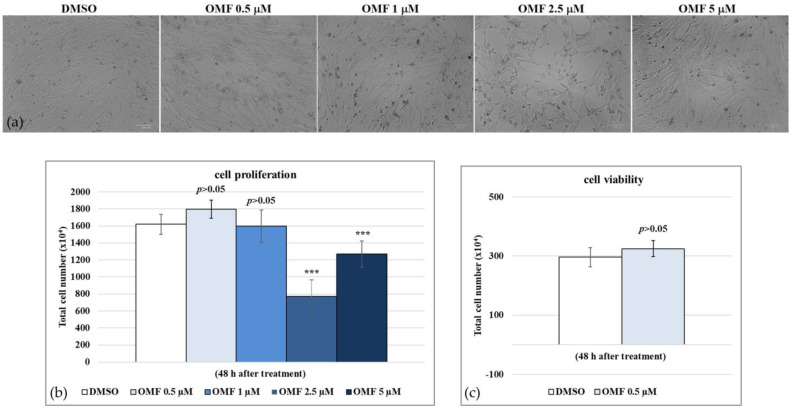
Determination of IC_50_ of OMF at various concentrations and development of dose–response curve for A72 cells. (**a**) A72 cells exposed to dimethyl sulfoxide (DMSO) (0.5 μM) or OMF at various doses (0.5, 1, 2.5, and 5 μM) at 48 h of treatment. (**b**) Dose–response curve of A72 cells exposed to DMSO (0.5 μM) or OMF at indicated doses (0.5, 1, 2.5, and 5 μM). At 48 h after treatment, A72 cells were tested using an MTT assay. (**c**) Cell viability was determined using TB staining and counted using a light microscope. Scale bar 100 μm. Significant differences between control and OMF-exposed cells were indicated by probability *p*. *** *p* < 0.001.

To examine the effect of OMF in CCoV infection in A72 cells, cell proliferation by 3-(4,5-dimethyl-2-thiazolyl)-2,5-diphenyl-2*H*-tetrazolium bromide (MTT) and cell viability using a TB exclusion test were assessed. First, half-maximal inhibitory concentrations (IC_50_) of OMF were identified and dose–response curves were developed after treatment of A72 cells with various concentrations of OMF (Figure 2a). Hence, A72 cells, in monolayer, were infected or not with CCoV at a multiplicity of infection (MOI) of five, and were exposed or not to OMF at various doses (0.5, 1, 2.5 and 5 μM), yielding the following groups: uninfected or infected cells, and OMF-exposed infected and uninfected cells. Cell growth inhibition was detected in A72 cells with an IC_50_ of approximately 2.5 μM OMF after 48 h of treatment (Figure 2b). OMF at 1 and 0.5 µM in A72 cells produced no significant differences in cell proliferation, as assessed using an MTT test (*p* > 0.05) (Figure 2b). Similar results were observed by analyzing cell viability using trypan blue (TB) staining (Figure 2c). Thus, these results demonstrated that OMF at a concentration of 0.5 μM did not significantly alter A72 cell viability or cell proliferation. Interestingly, the cell toxicity at 2.5 μM seemed to be high compared to 5 μM OMF in A72 cells (Figure 2a,b). This result was previously observed in bovine cells (MDBK) treated with OMF [17]. This nonmonotonic dose–response has been observed for a variety of compounds, including micronutrients, endocrine disrupting chemicals, and endogenous hormones, which typically bind nuclear receptors [19].

**Figure 3:**
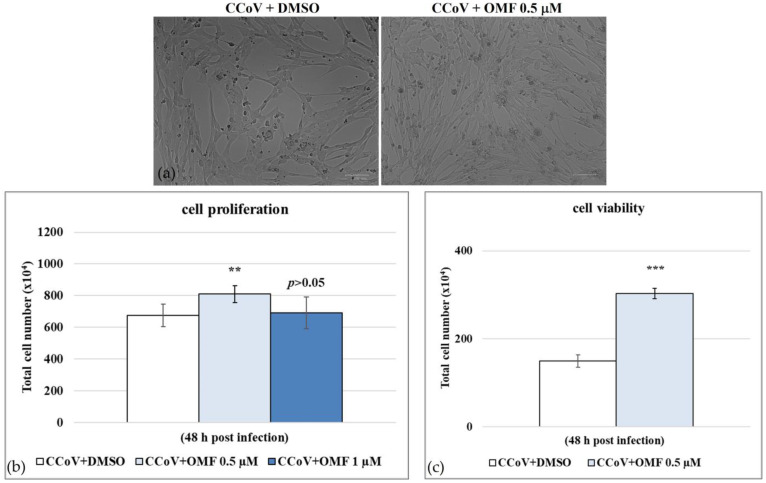
OMF reduced cell death during CCoV infection in A72 cells. (**a**) Cells infected with CCoV and exposed to OMF at various doses (0.5 and 1 μM) at 24 h after infection. (**b**) Dose–response curve of cells infected with CCoV, exposed to OMF (0.5 μM) for 48 h and tested using an MTT assay. (**c**) Dose–response curve of cells infected with CCoV, stained using TB and scored using a light microscope. Scale bar 100 μm. Significant differences between infected and OMF-exposed infected cells were indicated by probability *p*. ** *p* < 0.01 and *** *p* < 0.001.

During CCoV infection in A72 cells in the presence of OMF at 0.5 µM, cell proliferation (*p* < 0.01) (Figure 3a,b) and cell viability (*p* < 0.001) (Figure 3c) significantly increased. Hence, the concentration of OMF at 0.5 µM was selected to be utilized throughout the study. Our findings showed that during CCoV infection in A72 cells at the non-toxic dose of 0.5 µM, OMF significantly reduced cell death after 48 h of infection.

### 2.3. OMF Reduced Signs of Morphological Cell Death during CCoV Infection in A72 Cells

**Figure 4:**
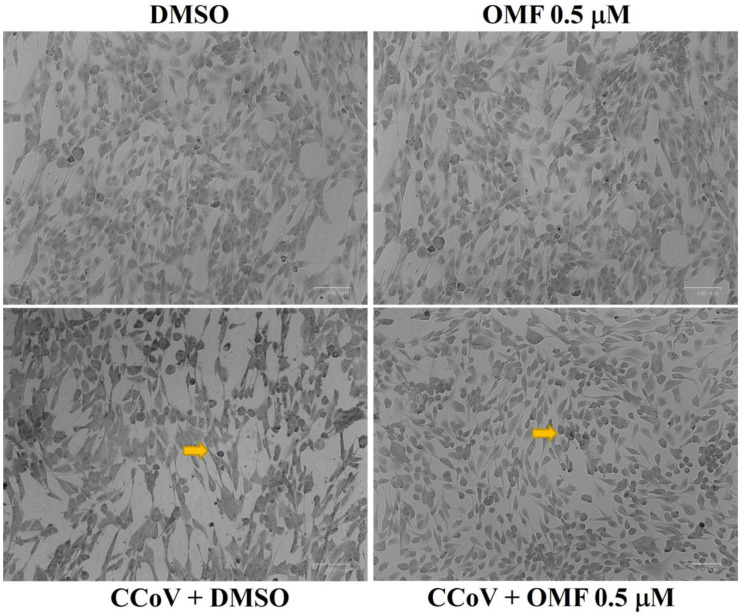
OMF diminished signs of morphological cell death during CCoV infection in A72 cells. Cells were infected with CCoV, exposed or not to OMF. After 48 h of infection, cells were observed under a light microscope after Giemsa staining. Comparing cells unexposed to OMF to control groups, photomicrographs revealed no morphological changes. In CCoV-infected groups, several cells showed signs attributable to apoptosis, such as pyknotic nuclei and nuclear fragmentation (arrow), whereas in OMF-treated infected cells, few signs of apoptotic cell death were noticed (arrow). Scale bar 100 μm.

To observe the influence of OMF during CCoV infection, A72 cell morphology was analysed using Giemsa staining and light microscopy at 48 h of infection. As displayed in Figure 4, the comparison between OMF-exposed uninfected cells and the control group showed no changes in morphology. An increase in intercellular spaces due to detachment from the culture plate were observed in unexposed infected cells. These features were accompanied by changes in morphology suggesting signs of apoptotic cell death, such as cellular shrinkage, pyknosis, and chromatin condensation (Figure 4, arrow). All cell death features were markedly diminished in CCoV-infected cells exposed to OMF (Figure 4, arrow). Overall, our findings demonstrated that OMF remarkably protected A72 cells during CCoV infection.

### 2.4. OMF Reduced Virus Yield during CCoV Infection in A72 Cells

To study the influence of OMF during CCoV infection in A72 cells, virus titer and viral cytopathic effects (CPE) were analysed for 72 h after infection.

**Figure 5:**
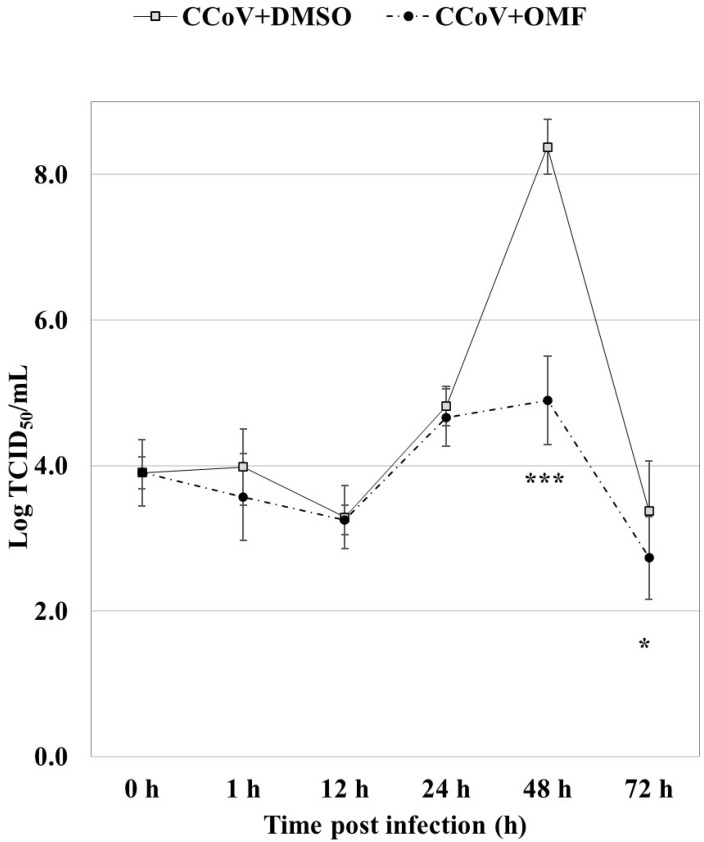
OMF diminished virus titer during CCoV infection in A72 cells. For viral growth curves, A72 cells were infected with CCoV at a MOI of five, exposed or not to OMF. At indicated times post infection, virus titers were assessed using RT-qPCR. Significant differences between CCoV-infected cells and OMF-treated infected cells were shown by probability *p*. *** *p* < 0.001 and * *p* < 0.05.

Remarkably, statistically significant (*p* < 0.001 and *p* < 0.05) declines in virus titer were detected via quantitative real-time RT-PCR (RT-qPCR) after 48 h and 72 h of infection, respectively, in cells exposed to OMF in CCoV infection (Figure 5). In addition, at 48 h after infection, CPE, identified by detachment from culture plates, was extensive in infected cells, whereas it noticeably lessened in OMF-exposed infected groups (Figure 4). Therefore, our findings revealed that OMF noticeably decreased virus yield and CPE during CCoV infection.

### 2.5. OMF Downregulated the Expression of AhR and NP during CCoV Infection

**Figure 6:**
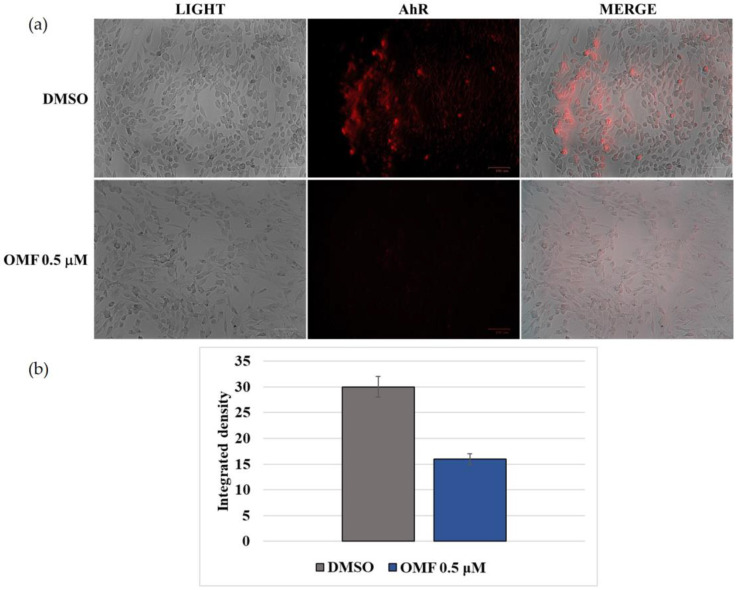
OMF downregulated the expression of AhR. (**a**) A72 uninfected group expressed AhR. OMF at a concentration of 0.5 μM markedly reduced the expression of AhR. Scale bar 100 µm. (**b**) Bars indicate the mean ratio produced from the integrated density in the expression of AhR assessed using ImageJ. Error bars correspond to standard error quantification.

To investigate the influence of OMF on AhR protein expression during CCoV infection in A72 cells, immunofluorescence (IF) staining was carried out. In the control group, represented by uninfected cells, we found that AhR was expressed [20], whereas in OMF-exposed cells, a downregulation of AhR protein expression was detected (Figure 6a). This result was proven using integrated density fluorescence measurement (Figure 6b).

**Figure 7:**
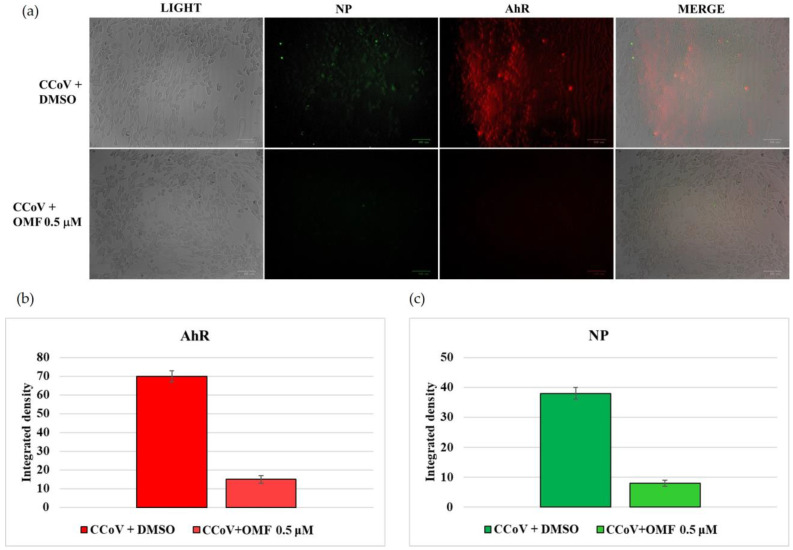
OMF decreased AhR and NP expression during CCoV infection in A72 cells. Cells were infected with CCoV at a MOI of five, and exposed or not to OMF; at 24 h after infection, IF staining to detect AhR (red fluorescence) and NP (green fluorescence) was carried out. (**a**) In OMF-exposed groups, the expression of AhR and NP was extremely decreased during CCoV infection. Scale bar 100 μm. (**b**) Bars correspond to the mean ratio obtained from the integrated density in the expression of AhR calculated using ImageJ. Error bars correspond to standard error quantification. (**c**) Bars correspond to the mean ratio obtained from the integrated density in the expression of NP calculated using ImageJ. Error bars correspond to standard error quantification.

Moreover, during CCoV infection exposed or not to OMF, the expression of AhR and NP was analysed using IF staining. After 24 h of infection, a noticeable reduction in both AhR and NP was detected in the presence of OMF (Figure 7a,b).

Taken together, these findings indicated that both NP and AhR expression were decreased by OMF.

## 3. Discussion

The need to find new antiviral products dramatically increased after the impact of COVID-19. So far, fungi have been particularly considered as a valuable source of antibiotic, antitumor, and immunomodulatory products [21,22]; however, recent investigations have shown that many fungal compounds possess remarkable antiviral properties [11,12], which deserve further assessment in view of integration with current drugs available to treat CoVs [23].

The search for potential chemotherapeutics is supported by insights regarding the structural aspects of virus proteins [24]. Indeed, computational approaches have confirmed that many fungal products could be effectively employed in therapy against SARS-CoV-2 by acting as protein inhibitors [25]. Pyranonigrin A, produced by *Penicillium thymicola*, was identified as a possible inhibitor of the main protease (Mpro) of the virus using docking and molecular dynamics simulation [26]. Moreover, fonsecin, a naphthopyrone produced by *Aspergillus fonsecaeus*, displayed a high binding affinity for the papaine-like protease of SARS-CoV-2 through interaction with the Tyr268 amino acid residue of the enzyme cavity [27]. Pyrrocidine A, a polyketide-amino acid-derivative from the endophytic fungus *Acremonium zeae*, and 18-methoxy-cytochalasin J were characterized as potent inhibitors of viral RNA-dependent RNA polymerase [28]. Finally, cyclosporine A, a renowned immunosuppressant also proposed for the treatment of hepatitis C [29] and MERS-CoV [30], was shown to be able to suppress replication of CoVs [31,32].

As a typical class of fungal secondary metabolites displaying a wide range of bioactive properties [14], the funicones have been recently characterized as having interesting antiviral properties. In this respect, deoxyfunicone was reported to be effective as an HIV-1-integrase inhibitor [33,34], and OMF was found to be able to reduce the infectivity of the hepatitis C virus [16]. Concerning the latter compound, in our previous studies, OMF induced a significant reduction in virus yield, and deeply inhibited bICP0 expression, the main regulatory protein in the lytic cycle of BoHV-1 [17].

In this study, OMF, at the non-toxic concentration of 0.5 μM, did not significantly increase cell death during CCoV infection, and weakened morphological cell death marks, which typically developed during CCoV infection in A72 cells [35,36]. Indeed, following in vitro infection, it has been shown that virus-induced apoptosis occurs in the presence of modulation in the levels of the sirtuin and FOXO family, which are proteins involved in cell damage due to apoptosis or oxidative stress [37].

As previously detected in BoHV-1 infection [17], herein, during CCoV infection, OMF induced a significant decrease in virus yield, which occurred in the presence of a downregulation of AhR, indicating AhR’s involvement in OMF’s antiviral action. These findings are in agreement with recent evidence showing the involvement of AhR in both human and animal coronaviruses infections [38,39,40,41]. Indeed, AhR inhibition induces a decrease in the expression of angiotensin converting enzyme 2 receptors, accompanied by an inhibition of SARS-CoV-2 infection in human cells [20,40,41]. In addition, AhR stimulates the enzyme indolamine 2,3 dioxygenase, which is involved in the production of kynurenine starting from tryptophan. These regulatory actions act cell growth by inducing apoptosis [41]. Herein, following in vitro CCoV infection, an antiviral activity, accompanied by a reduction in morphological apoptotic features, was likely due to OMF, as its aromatic nature probably inhibits AhR.

Conversely, during BoHV-1 infection, OMF provokes an increase in the expression of AhR [17]. This conflicting phenomenon emphasizes the timing of AhR induction for regulating the balance between immunopathology and antiviral defensive immunity [42]. Indeed, it has been described that AhR activation promotes the replication of herpesviruses, including cytomegalovirus, herpes simplex I (HSV-1), HSV-II, and BoHV-1 [43,44]. In particular, several mice died due to herpes encephalitis following HSV-1 infection when AhR was up-regulated before infection, whereas, when stimulation of AhR occurred subsequent to HSV-1 infection, herpetic pathology was reduced.

In conclusion, in the current scenario of emerging pathogenic CoVs such as SARS-CoV2, our findings highlight the ability of OMF to reduce CCoV infection. Moreover, we also show the convenience of exploring the potential anti-viral properties of natural molecules, such as OMF, using an in vitro system; this represents an effective approach for studying CoVs without the manipulation of hazardous viruses.

## 4. Materials and Methods

### 4.1. Production and Isolation of OMF

Mycelial plugs from actively growing cultures of *T. pinophilus* LT6 were inoculated in 1 L-Erlenmayer flasks containing 500 mL of potato dextrose broth (PDB, Himedia, West Chester, PA, USA). The cultures were kept in a stationary phase in darkness at 25 °C. After 21 days, the liquid phase (3 L) was separated via filtration at 0.45 µm, and the culture filtrate was concentrated via lyophilization until it was reduced to 1/10 of the starting volume. The latter was extracted three times with the same volume of chloroform, as previously reported [18]. The crude extract (43.4 mg) was submitted to fractionation via column chromatography (1.0 × 30 cm i.d.) on silica gel (Kieselgel 60, 0.063–0.200 mm, Merk, Darmstadt, Germany), and eluted with CHCl_3_/*iso*-PrOH (95:5, *v/v*). Six homogeneous fraction groups were collected (A, 2.1 mg; B, 9.2 mg; C, 4.1 mg; D, 2.3 mg; E, 4.1 mg; and F, 8.2 mg). The residue of the second fraction was purified using thin layer chromatography (TLC) on silica gel (Kieselgel 60, F254, 0.25 mm, Merk) eluted with CHCl_3_/*iso*-PrOH (95:5, *v*/*v*), yielding OMF (4.1 mg). It was identified by comparing NMR data with previously reported data [18].

### 4.2. Cell Cultures and Virus Infection

A72 cells (canine fibrosarcoma cell line) were cultivated in Dulbecco’s modified Eagle’s minimal essential medium (DMEM) at 37 °C and 5% CO_2_ [36,37]. The strain S/378 of CCoV type II was utilized throughout the study. For virus stocks growth and virus titration, A72 cells were utilized [36].

OMF was first dissolved in DMSO, and then added to the medium at final doses of 0.5, 1, 2.5, and 5 μM.

A72 cells, in monolayers, were infected or not with CCoV, at a MOI of five, and exposed or not to OMF (0.5, 1, 2.5, and 5 μM), to obtain four groups: CCoV uninfected or infected cells, and OMF-exposed infected and uninfected groups. Cells were incubated 1 h after adsorption at 37 °C, and treated at 0, 1, 12, 24, 48, and 72 h after infection. The virus was in culture medium for the duration of the experiment.

### 4.3. Cell Proliferation

Cell proliferation evaluation was assayed via MTT assay [17,45,46,47]. In brief, A72 cells cultured in 96-well plates were infected or not with CCoV at a MOI of five, and exposed or not to OMF (0.5, 1, 2.5, and 5 μM) to obtain four groups: uninfected or infected cells, and OMF-exposed infected and uninfected cells, and then incubated. After 48 h of infection, MTT assays were performed. Results were the mean ± S.D. of three separate experiments conducted in duplicate.

### 4.4. Cell Viability

Cell viability was evaluated using a TB (Sigma-Aldrich, Milan, Italy) exclusion test [17]. A72 cells, in monolayer, were infected or not with CCoV at a MOI of five, exposed or not to OMF at 0.5 μM, incubated for 48 h, and processed as previously reported [17]. Cell viability was then evaluated as a percentage of living cells over the total cell number. Results were reported as the mean ± S.D. of three separate experiments conducted in duplicate.

### 4.5. Examination of Cell Morphology

To study the morphology of A72 cells, Giemsa staining followed by light microscopy examination was utilised [17,48]. A72 cells were infected or not with CCoV at a MOI of five, exposed or not to OMF (5 µM), and incubated at 37 °C. At 48 h p.i., Giemsa staining was carried out, and light microscopy analysis was performed using a ZOE Cell Imager (Bio-Rad Laboratories, Segrate, Milan, Italy). To identify cellular death characteristics, criteria previously reported were used [49,50,51].

### 4.6. IF Staining

A72 cells were infected or not with CCoV at a MOI of five, and exposed or or not to OMF (0.5 µM). At 24 h p.i., IF staining was assessed [17,52] using antibodies dissolved in 5% bovine serum albumin-TBST: anti-AhR (Sigma-Aldrich) (1:250); anti-NP monoclonal mouse, MAB 938 (The Native Antigen Company, Kidlington, UK); Alexa Fluor 488 goat anti-mouse (Thermo Fisher Scientific, Waltham, MA, USA) (1:1000); and Texas Red goat anti-rabbit (Thermo Fisher Scientific) (1:100). Microscopic study and photography were undertaken using a ZOE Fluorescent Cell Imager (Bio-Rad Laboratories). Fluorescence signals of images from microscopy were quantified using ImageJ software (version 1.53a, National Institutes of Health, Bethesda, MD, USA).

### 4.7. Virus Production

A72 cells were infected or not with CCoV at a MOI of five, exposed or not to OMF, incubated at 37 °C, and processed after 0, 1, 12, 24, 48, and 72 h p.i. using quantitative real-time RT-PCR (RT-qPCR) for CCoV quantification. In addition, CPE was examined. Therefore, at indicated times p.i., cells were analysed using a light microscope, as previously reported [17,36].

### 4.8. Viral Nucleic Acids Extraction Procedures

Nucleic acids were isolated from the samples (200 µL cell supernatant) using an automatic extraction system (King Fisher Flex) employing the MagMax Viral Pathogen kit (all Thermo Fisher Scientific, Waltham, MA, USA). DMEM was utilized as a negative process control and elution was carried out in 60 µL.

### 4.9. Real-Time RT-PCR for CCoV Quantification

RT-qPCR was carried out to evaluate the titer of CCoV II during infection in A72 cells with or without OMF. Detection was carried out at various times post infection (1, 12, 24, 48, and 72 h) using a QuantStudio 5 Real-Time PCR instrument (Thermo Fisher Scientific). For each sample, the reaction (25 µL) contained: 5 µL of nucleic acids, 1× AGPATH reaction mix, 1× reverse transcriptase-PCR enzyme mix (Thermo Fisher Scientific), 1 µL (10 µM) of each primer (CCoV-For 5’-TTGATCGTTTTTATAACGGTTC-TACAA-3’, CCoV-Rev 5’-AATGGGCCATAATAGCCACATAAT-3’) and 1 µL (6 µM) of probe CCoV-P (FAM-5’-ACCTCAATTTAGCTGGTTCGTGTATGGCATT-3’-TAMRA) [53]. The thermal profile was reverse transcription for 30 min at 42 °C, followed by 15 min at 95 °C, 40 cycles of 15 s at 95 °C, and 60 s at 60 °C. A standard curve was used to quantify CCoV in the samples; it was built by analysing serial dilutions of the extracted virus (from 3.5 × 10^9^ to 3.5 × 10^4^ TCID_50_/mL) and plotting the Log TCID_50_/mL versus the Ct number. The presence of PCR inhibitors was evaluated for each sample as previously described elsewhere [54], by adding murine norovirus as an external process control (EPC) [55].

### 4.10. Statistical Analysis

Data are shown as mean ± S.D. GraphPad InStat Version 3.00 for Windows 95 (GraphPad Software, San Diego, CA, USA) was used to perform one-way ANOVA with Tukey’s post-test; *p* < 0.05 was considered statistically significant.

## Data Availability

The data that support the findings of this study are available from the corresponding author upon reasonable request.
